# Case Report: Infectious prophylaxis in hematological malignancies

**DOI:** 10.3389/fonc.2023.1163175

**Published:** 2023-05-01

**Authors:** Mauro Passucci, Chiara Masucci, Francesca Paoletti, Claudia Ielo, Alessandro Costa, Ida Carmosino, Emilia Scalzulli, Maurizio Martelli, Giuseppe Gentile, Massimo Breccia

**Affiliations:** ^1^ Department of Translational and Precision Medicine, Policlinico Umberto I, La Sapienza University of Rome, Rome, Italy; ^2^ Hematology Unit, Businco Hospital, ARNAS G. Brotzu, Cagliari, Italy

**Keywords:** HBV reactivation, myelofibrosis, ruxolitinib, tenofovir, prophylaxis

## Abstract

Patients with hematological malignancies and past serological evidence of hepatitis B are at risk for HBV reactivation. In myeloproliferative neoplasms, continuous treatment with the JAK 1/2 inhibitor ruxolitinib confers a moderate risk of reactivation (1-10%); nevertheless, no prospective randomized data are available to strongly recommend HBV prophylaxis in these patients. Here, we report a case of primary myelofibrosis and past serological evidence of HBV infection, treated with ruxolitinib and concomitant lamivudine, developing HBV reactivation due to premature withdrawal of prophylaxis. This case underlines the potential need for persistent HBV prophylaxis in the setting of ruxolitinib treatment.

## Introduction

Patients affected by hematological malignancies (HMs) are at risk for hepatitis B virus (HBV) reactivation, with higher vulnerability for those who have surface antigen (HBsAg) and lower susceptibility in case of isolated anti-core antibodies (HBcAb) (1). Both the underlying diseases and anti-cancer therapies play a crucial role in the development of immunosuppression allowing HBV reactivation. Patients treated with chemo-immunotherapy (including anti-CD20 monoclonal antibodies) or recipients of hematopoietic stem cell transplant are considered at high risk because > 10% of these cases experience HBV reactivation, recommending prohylaxis (2). Novel target therapies have shown considerable efficacy in HMs until progression or intolerance but infections can occur to a significant level, in particular in patients receiving JAK inhibitors (3). However, the role of HBV prophylaxis in this setting is still debated. Here we report a case of overt-primary myelofibrosis (overt-PMF) and past HBV infection, treated with the JAK inhibitor ruxolitinib, who experienced HBV reactivation due to non-adherence to primary prophylaxis with lamivudine.

## Case presentation

**Figure 1:**
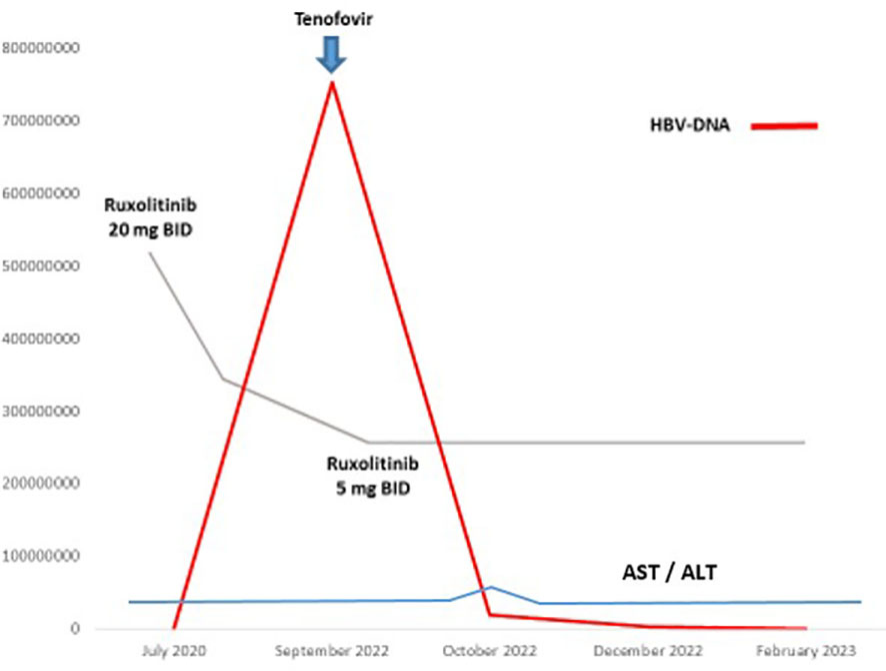
HBV-DNA levels during treatment (UI/ml, red line).

An 82-year-old man was diagnosed with IPSS intermediate-2 risk overt-PMF in July 2020. At baseline the patient presented splenomegaly (> 5 cm below costal margin) and systemic symptoms (4), so we decide to start ruxolitinib at the recommended dose of 20 mg BID soon after diagnosis was made. Virological screening performed before starting ruxolitinib revealed HBcAb in the absence of HBsAg and its antibodies. No circulating HBV-DNA and HCV-RNA copies were detected: concomitant prophylaxis with lamivudine 100 mg QD was started. Combined treatment was well tolerated, except for a transient moderate amylase increase in March 2022. A complete splenic volume response was achieved according to IWG criteria (5) without transfusion requirement. In September 2022, at routine blood test examination, a virological monitoring revealed persistent anti-core antibodies with the appearance of HBsAg and anti-HBs. Even in the absence of symptoms and abnormal liver function, plasma HBV-DNA test was performed, revealing 754.790.947 UI copies/mL. On further investigation, the patient revealed he discontinued lamivudine in May 2022 without medical indication. According to infectious disease consulting, specific anti-HBV treatment with tenofovir alafenamide (TAF) 25 mg QD was started. Considering the persistent splenic response, ruxolitinib was de-escalated to 5 mg BID to prevent further liver damage. HBV-DNA trend during treatment is reported in **Figure 1**. Only a mild and transient increase in liver transaminases related to combined treatment was revealed after 1 month associated with a progressive decline in HBV-DNA copy level. At the last follow-up in February 2023, HBV-DNA copies were 221.000 UI/ml without signs of active hepatitis or hematologic disease relapse.

## Discussion

T-cell depletion/dysfunction and worse anti-viral immunity are often encountered in the management of hematological malignancies, especially in patients with lymphoma (about 40% of patients with anti-HBc without HBsAg, anti-HBs and HBV-DNA) or recipients of HSCT. Therefore, HBV reactivation in this setting is not quite a rare occurrence and can potentially be harmful in terms of morbidity and mortality (6, 7). Despite the availability of consensus guidelines, risk stratification for HBV reactivation and related prophylactic strategies are not fully established (8). Currently, screening with HBsAg, anti-HBc and HBV-DNA is recommended at baseline for all patients undergoing potentially immunosuppressive treatment for HMs. Moreover, latest guidelines of the European Association for the Study of the Liver (EASL) recommend prophylaxis with the third-generation nucleoside analogues entecavir (ENT) or tenofovir (disoproxil fumarate TDF/alafenamide TAF) in all patients with evidence of chronic infection (i.e., HBsAg without active hepatitis). Patients with serological evidence of past HBV infection (i.e., isolated anti-HBc antibodies) should receive prophylaxis with either lamivudine (LAM) or ENT/TDF-TAF only in case of moderate or high risk of reactivation (9). Ruxolitinib confers a moderate risk of HBV reactivation (1-10%) (10). In myeloproliferative neoplasms (MPNs) like MF and polycythemia vera, ruxolitinib inhibits the JAK/STAT pathway, acts on dendritic cells, CD4 + T and Natural Killer cells differentiation or function, inhibiting the production of cytokines such as IFNγ and TNFα, with consequent impairment of an adequate infection control (11). All patients should be screened for HBsAg, anti-HBc, anti-HBs and HBV-DNA (if anti-HBc positive) before starting ruxolitinib (12); vaccination is recommended in HBV-naïve patients. However, the role of prophylaxis in this intermediate-risk category is not fully understood. Patients with active hepatitis (HBsAg with increased transaminases) were excluded from larger clinical trials with ruxolitinib and events of HBV reactivation during ruxolitinib continuous treatment are limited to case reports (13). Lamivudine prophylaxis is often used in patients with past serological HBV evidence; according to other authors, a risk-adapted strategy based on high potency entecavir or tenofovir could be offered to patients undergoing prolonged immunosuppression (i.e., more than 12 months (8, 10)). However, both these strategies are not validated by randomized prospective trials. Tenofovir alafenamide (TAF) is reported as effective for both prophylaxis and treatment and is safer than tenofovir disoproxil fumarate (TDF), due to the lower renal and bone toxicity (14).

## Conclusions

In our case, combined treatment consisting of reduced dose of ruxolitinib and tenofovir alafenamide was well tolerated without concerns for hematologic disease control. In our opinion, this case highlights the potential deep immunosuppression during ruxolitinib treatment and the usefulness of HBV serological screening at baseline. Prospective data are mandatory to establish the possible role of continuous HBV prophylaxis in this setting.

## Data availability statement

The original contributions presented in the study are included in the article/supplementary material. Further inquiries can be directed to the corresponding author.

## Ethics statement

Written informed consent was obtained from the individual(s) for the publication of any potentially identifiable images or data included in this article.

## Author contributions

Manuscript writing: MP, CM, FP, AC, CI. Review and editing: MB, GG, MM, ES, IC. Figures: MP. All authors contributed to the article and approved the submitted version.

## References

[1] Chang Y Jeong SW Jang JY. Hepatitis b virus reactivation associated with therapeutic interventions. Front Med (Lausanne). (2022) 8:770124. doi: 10.3389/fmed.2021.770124 35096867 PMC8795508

[2] Law MF Ho R Cheung CK Tam LH Ma K So KC. Prevention and management of hepatitis b virus reactivation in patients with hematological malignancies treated with anticancer therapy. World J Gastroenterol (2016) 22(28):6484–500. doi: 10.3748/wjg.v22.i28.6484 PMC4968128 27605883

[3] Reinwald M Boch T Hofmann WK Buchheidt D. Risk of infectious complications in hemato-oncological patients treated with kinase inhibitors. biomark Insights (2015) 10(Suppl 3):55–68. doi: 10.4137/BMI.S22430 PMC4841329 27127405

[4] Yi CA Tam CS Verstovsek S. Efficacy and safety of ruxolitinib in the treatment of patients with myelofibrosis. Future Oncol (2015) 11(5):719–33. doi: 10.2217/fon.14.272 PMC4920055 25757677

[5] Tefferi A Cervantes F Mesa R Passamonti F Verstovsek S Vannucchi AM. Revised response criteria for myelofibrosis: international working group-myeloproliferative neoplasms research and treatment (IWG-MRT) and European LeukemiaNet (ELN) consensus report. Blood (2013) 122(8):1395–8. doi: 10.1182/blood-2013-03-488098 PMC4828070 23838352

[6] Marcucci F Mele A Spada E Candido A Bianco E Pulsoni A. High prevalence of hepatitis b virus infection in b-cell non-hodgkin’s lymphoma. Haematologica (2006) 91(4):554–7. 16585021

[7] Hwang JP Barbo AG Perrillo RP. Hepatitis b reactivation during cancer chemotherapy: an international survey of the membership of the American association for the study of liver diseases. J Viral Hepat (2015) 22(3):346–52. doi: 10.1111/jvh.12305 PMC4833504 25220947

[8] Sarmati L Andreoni M Antonelli G Arcese W Bruno R Coppola N. Recommendations for screening, monitoring, prevention, prophylaxis and therapy of hepatitis b virus reactivation in patients with haematologic malignancies and patients who underwent haematologic stem cell transplantation-a position paper. Clin Microbiol Infect (2017) 23(12):935–40. doi: 10.1016/j.cmi.2017.06.023 28668466

[9] European Association for the Study of the Liver Electronic address, e. e. e European Association for the Study of the, L. EASL 2017 clinical practice guidelines on the management of hepatitis b virus infection. J Hepatol (2017) 67(2):370–98. doi: 10.1016/j.jhep.2017.03.021 28427875

[10] Wang B Mufti G Agarwal K. Reactivation of hepatitis b virus infection in patients with hematologic disorders. Haematologica (2019) 104(3):435–43. doi: 10.3324/haematol.2018.210252 PMC6395346 30733266

[11] Sjoblom M Chtioui H Fraga M Stalder G Grandoni F Blum S. Hepatitis b reactivation during ruxolitinib treatment. Ann Hematol (2022) 101(9):2081–6. doi: 10.1007/s00277-022-04851-6 35488090

[12] Sandherr M Hentrich M von Lilienfeld-Toal M Massenkeil G Neumann S Penack O. Antiviral prophylaxis in patients with solid tumours and haematological malignancies–update of the guidelines of the infectious diseases working party (AGIHO) of the German society for hematology and medical oncology (DGHO). Ann Hematol (2015) 94(9):1441–50. doi: 10.1007/s00277-015-2447-3 PMC4525190 26193852

[13] Sant’Antonio E Bonifacio M Breccia M Rumi E. A journey through infectious risk associated with ruxolitinib. Br J Haematol (2019) 187(3):286–95. doi: 10.1111/bjh.16174 31468506

[14] Inada K Kaneko S Kurosaki M Yamashita K Kirino S Osawa L. Tenofovir alafenamide for prevention and treatment of hepatitis b virus reactivation and *De novo* hepatitis. JGH Open (2021) 5(9):1085–91. doi: 10.1002/jgh3.12636 PMC8454476 34584979

